# Cyclopedia of the Diseases of Children

**Published:** 1889-08

**Authors:** 


### Book Reviews.

**Cyclopedia of the Diseases of Children—Medical and Surgical.** The articles written especially for the work by American, British, and Canadian authors. Edited by John M. Keating, M. D. Vol. I. Illustrated; 8vo, pp. xiii., 992. Philadelphia: J. B. Lippincott Company. 1889.

It is almost surprising in these days of encyclopedic medical literature, that the department of pediatrics should have escaped so long. It is fortunate, however, that it has finally been taken up by such competent hands, for, in the work before us, both editor and publishers seem to have vied with each other in the production of a treatise of extreme excellence. The authors, too, have been chosen with discretion, and have written understandingly in their several branches.

As is already known through the announcements that have heralded the forthcoming of this work for several months, it is proposed to publish it in four handsome imperial octavo volumes of about 800 pages each, each to be carefully indexed, and the set to be completed in about a year. The volume at hand is the first of the series, and it is not an exaggeration to say that the publishers have more than fulfilled their promises in its production. It is printed on No. 1 book paper, in clear-faced handsome type, and its illustrations, as a whole, are most excellent in character. So much for the work in general.

Coming to the particulars of the present volume, the first thing to attract attention is the introductory chapter, written by that most competent, erudite, and scholarly author, Dr. Abraham Jacobi, of New York. This chapter is an essay in and of itself, and should be carefully studied by every man interested in the management of children; and who is not?

Dr. Jacobi says (page 2) that "Pediatrics . . . is no specialty in the common acceptation of the term. It does not deal with an organ, but with the entire organism at the only period which presents the most interesting features to the student of biology and medicine. Infancy and childhood are links between conception and death, between the fetus and the adult. The latter has attained a certain degree of invariability. His physiological labor is reproduction, that of the young is both reproduction and growth."

Again, " . . . pediatrics does not deal with miniature men and women, with reduced doses and the same class of diseases in smaller bodies, but . . it has its own independent range and horizon, and gives as much to general medicine as it has received from it." True words, that deserve to be pondered.

The first general subject is naturally enough on the Anatomy of Children, and is written by Dr. George McClellan. This runs through forty pages, and is beautifully illustrated by photographs from life, frozen sections, and by drawings of dissections.

The article on Diagnosis, by Dr. James Finlayson, is one of the greatest importance here as elsewhere in medicine, and could not have been more satisfactorily handled. Here the student and young practitioner will find invaluable hints and instruction for their guidance in the initial management of their young patients; for this is a branch of practice that is especially beset with difficulties for the beginner.

The chapter on Outlines of Practical Bacteriology, by Edward O. Shakespeare, M. D., is a most interesting and learned discourse upon this important subject, but we hardly appreciate the necessity of presenting it in a work of this kind. It, too, is well illustrated, which adds much to the attractiveness and understanding of the text.

Maternal Impressions, by Dr. William C. Dabney, is a philosophical essay on the subject, but we think it might properly appear in a work on obstetrics.

A chapter that deserves especial study is that on The Care of the Child at and Immediately after Birth, in Health and Disease, by Dr. R. A. F. Penrose. We are glad to read here: "We apply this ligature (to the cord) . . . . as soon as the function of respiration becomes fully established—that is, when the child breathes well, and, in most instances, has cried lustily" (p. 237). This seems to us a most sensible statement on a point where there is some division of opinion, but, all in all, it is about as safe practice as any. Dr. Penrose's instructions for dealing with the asphyxiated newly-born are clear, and bear the imprint of experience.

Infant-Feeding and Weaning are always vexed questions, but they receive most intelligent treatment at the hands of Dr. T. M. Rotch. The question of proper nutriment lies at the very foundation of the vast majority of the diseases of infancy, and cannot be too carefully studied by all who are called upon to minister to these little patients. The per cent. of infant mortality from improper feeding is truly appalling, and whoever contributes to its reduction by even a minute fraction is a philanthropist.

A closely-allied subject to the last is that of Wet-Nurses, and this has been dealt with by a most competent observer, Dr. William H. Parish. Though the chapter is short, it is full of practical thought, and its treatment is able.

Diet after Weaning, by Dr. Samuel S. Adams, is a subject akin to the preceding ones, and so very properly follows them.

The Nursing of Sick Children, by Miss Catherine Wood, is written by a person of experience in this direction, and one whose heart is in her work; else she could not discourse so ably, even touchingly, upon the subject.

The subject of Nursery Hygiene is of great importance in these days, when so many mothers relegate the care of their children to the nurse and the nursery. Dr. L. M. Yale gives some most valuable suggestions on this question, and no one is more competent to speak thereon than he.

Dentition and Puberty form the subjects of the next two chapters, written by Drs. John Dorning and Thomas More Madden, respectively. They are replete with teachings of value, and deserve the careful examination of the student to be appreciated. This closes the general subjects treated of in Part I.

We have not been able to treat of all the titles in this part, but have merely attempted to mirror the most important features thereof.

In Part II. the special consideration of the several diseases belonging to infancy and childhood begins, and the first group includes Fevers and Miasmatic Diseases.

We cannot at this time, nor does it seem necessary to, consider all the various titles of this part of the book in detail. It must be regarded as sufficient for us to call attention to the general fact, and let each subscriber to the series find out for himself that he has not invested his money vainly.

It were not possible to obtain perfection in the publication of such a comprehensive work as this cyclopedia contemplates, but we are of the opinion that the editor, authors, and publishers have as nearly approached thereunto as can be done in a first attempt. The latest thoughts on many of the subjects will here be found, and every teacher and practiser of pediatrics must equip his book-shelves with this great work for reference and study.

